# The Effects of 1-Hz rTMS on Emotional Behavior and Dendritic Complexity of Mature and Newly Generated Dentate Gyrus Neurons in Male Mice

**DOI:** 10.3390/ijerph17114074

**Published:** 2020-06-08

**Authors:** Marco Cambiaghi, Rosalia Crupi, Erick Larios Bautista, Amir Elsamadisi, Wasib Malik, Helen Pozdniakova, Zhiyong Han, Mario Buffelli, Fortunato Battaglia

**Affiliations:** 1 Department of Neurosciences, Biomedicine and Movement Sciences-University of Verona, 37134 Verona, Italy; marco.cambiaghi@univr.it (M.C.); mario.buffelli@univr.it (M.B.); 2 Department of Chemical, Biological, Pharmaceutical and Environmental Sciences, University of Messina, 98125 Messina, Italy; rcrupi@unime.it; 3 Department of Medical Sciences, Neurology and Psychiatry, Hackensack Meridian School of Medicine, Seton Hall University, Nutley, NJ 07110, USA; erick.lariosbautista@student.shu.edu (E.L.B.); amir.elsamadisi@student.shu.edu (A.E.); wasib.malik@student.shu.edu (W.M.); helen.pozdniakova@student.shu.edu (H.P.); zhiyong.han@shu.edu (Z.H.)

**Keywords:** 1-Hz rTMS, neurogenesis, brain stimulation, dentate gyrus, dendritic complexity

## Abstract

Low-frequency repetitive transcranial magnetic stimulation (1-Hz rTMS) is a promising noninvasive tool for the treatment of depression. Hippocampal neuronal plasticity is thought to play a pivotal role in the pathophysiology of depressive disorders and the mechanism of action of antidepressant treatments. We investigated the effect of 1-Hz rTMS treatment on hippocampal dentate gyrus structural plasticity and related emotional behaviors modifications. Experimentally, adult male mice received either five days of 1-Hz rTMS or Sham stimulation. After stimulation, the mice underwent a battery of tests for anxiety-like and depression-like behaviors. We also tested the effect of treatment on mature and newly generated granule cell dendritic complexity. Our data showed that 1-Hz rTMS induced structural plasticity in mature granule cells, as evidenced by increased dendritic length and number of intersections. However, the stimulation did not increase the proliferation of the dentate gyrus progenitor cells. On the contrary, the stimulated mice showed increased dendritic complexity of newly generated neurons. Moreover, 1-Hz rTMS resulted in antidepressant-like effects in the tail suspension test, but it did not affect anxiety-like behaviors. Therefore, our results indicate that 1-Hz rTMS modulates dentate gyrus morphological plasticity in mature and newly generated neurons. Furthermore, our data provide some evidence of an association between the antidepressant-like activity of 1-Hz rTMS and structural plasticity in the hippocampus.

## 1. Introduction

Major depressive disorders (MDD) are highly prevalent medical conditions [1] that have a significantly negative impact on quality of life [2]. Since the available pharmacotherapies are effective in 50–60% of MDD patients and may have significant side effects [3,4,5], there has been a growing interest in the development of nonpharmacological methods to treat depression. Repetitive transcranial magnetic stimulation (rTMS) is a brain stimulation intervention that noninvasively modulates brain circuitries. High-frequency (HF) rTMS (5–25 Hz) has antidepressant efficacy and it has become an established treatment option for drug-resistant depression [6]. The prevailing evidence suggests that low-frequency (LF) stimulation (1-Hz) applied to the right prefrontal cortex not only has clinical efficacy but also a better safety profile [7,8]. The mechanisms underlying the therapeutic effect of 1-Hz rTMS have not been fully characterized, although there are evidences indicating that it might be linked to Long-Term Potentiation/Long-Term Depression (LTP/LTD)-like plasticity that outlasts the period of stimulation [9]. Studies of patients and in animal models indicate that depression is associated to abnormal morphological plastic changes such hippocampal atrophy [10,11,12], altered hippocampal dendritic morphology and dentate gyrus (DG) adult neurogenesis [13,14]. Such morphological changes may induce clinically relevant functional changes in the hippocampus [15]. Currently, it has been established that DG adult neurogenesis is modulated by electroconvulsive treatment (ECT) [16], vagal nerve stimulation [17], deep brain stimulation [18], chronic selective serotonin reuptake inhibitors (SSRIs) treatment [19], and HF-rTMS treatment [20]. However, far less is known about the effect of 1-Hz rTMS treatment on brain morphology and behavior. In addition, the quantitative morphological analysis of dendritic architecture of mature and newly generated granule cells in the DG after rTMS treatments has not yet been investigated. In the DG, newly generated neurons are mostly located in the inner layer of the granule cell layer (GCL) while mature granule cells are located in the outer GCL [21]. The changes in dendritic branching of mature and newly generated hippocampal granule cells are closely related to depression, and understanding the factors that cause such changes is important in understanding the mechanism of action of 1-Hz rTMS [22].

Therefore, the present study was conducted to evaluate whether 1-Hz rTMS treatment promotes hippocampal DG plasticity and modulates emotional behaviors in male mice. We assessed the effect of a stimulation protocol on the proliferation of hippocampal progenitor cells and on the dendritic complexity of both mature and newly generated granule cell in the DG.

## 2. Methods

### 2.1. Animals

A total of 32 129/SvEv male mice (7–9 week old) were purchased from Taconic (Germantown, NY, USA). The 129/SvEv mouse strain is prone to anxiety [23] and shows longer immobility during behavioral despair tests [24]. Mice were provided with food and water, available ad libitum, and maintained on a 12:12 light/dark artificial cycle (lights on at 7:00 h). Mice were tested between 10:00 a.m. and 12:00 a.m. according to protocol approved by the Animal Care and Use Committee of the University of Verona (CIRSAL) and authorized by the Italian Ministry of Health (n. 718/2019-PR).

### 2.2. Experimental Procedures

Mice were distributed into two groups: (a) 1-Hz rTMS treatment group, (b) Sham stimulation mice. rTMS was applied in aware animals using a MagStim Rapid stimulator equipped with a 25 mm figure-of-eight rodent coil (The MagStim Company, Whitland, Dyfed, UK). The stimulation intensity just below the motor threshold value, estimated as visible contraction of upper limbs in all treated animals (50 ± 3.7% of the stimulator output). Resting Motor Threshold (RMT) was determined by decreasing stimulator output by 5% until movement vanished and then increasing in 1% increments until they appeared in at least 50% of 10 pulses total. We adjusted the coil positioning for achieving best possible responses.

**Figure 1:**
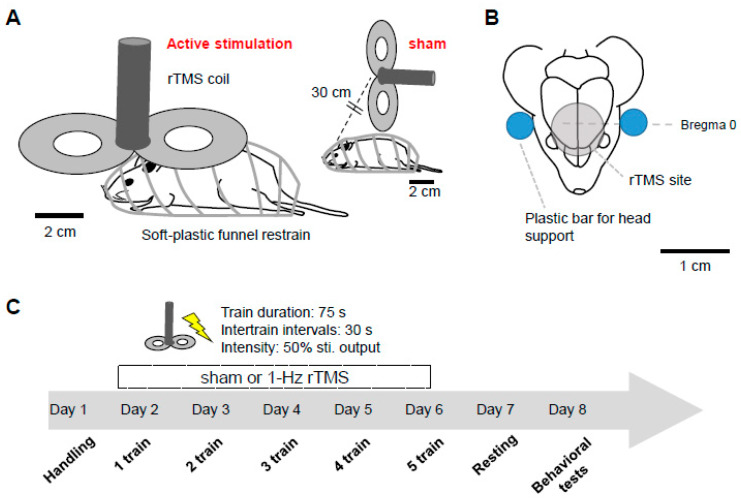
Stimulation procedure and experimental design. (**A**) Schematics of the stimulation procedure showing the awake restrained mouse during stimulation and the stimulated area (**B**). Head movements were minimized by using two plastic bars (right). All the analyses were performed in Sham and 1-Hz rTMS groups after a 5 days stimulation protocol. Behavioral tests were run in a random order twenty-four hours after the final rTMS session. (**C**). 1-Hz rTMS: low-frequency repetitive transcranial magnetic stimulation

For the 1-Hz treatment, the coil was kept very close to the mouse head (~0.5 cm distance between the skin and the coil, held in place by a dedicated support), with the center of the coil right above the frontal area (with an angle of 90 degrees with respect to the animals’ head). During the sham stimulation, the coil was not oriented toward the mouse head and it was positioned at about 30 cm from the mouse head. We delivered 1-Hz stimulation trains (75 s duration, 75 stimuli) separated by 30 s intervals. On day 1, mice were restrained in a plastic funnel for 5 s to familiarize with the experiment. In order to minimize the head’s movement, two plastic bars were used. The stimulation took place during five consecutive days with a progressive increase in stimulation trains (1 train on day 1, 5 trains on day 5). The animals were tested twenty-four hours after the final rTMS session (Figure 1). Because of the effect of repeated handling and stress on hippocampal structural plasticity and neurogenesis, we used a stimulation protocol with a smaller amount of stimuli compared with protocols used in a clinical setting.

### 2.3. Emotional Behaviors

All behavioral tests were videotaped and manually scored by an experimenter blind to the experimental conditions.

*Open Field Test (OFT).* We performed two different experiment. First, the animals (*n* = 10 each group) were placed in the center of an open field (50 cm × 50 cm) and their exploration of the field was assessed for 5 min. The field was divided into 16 identical squares by black lines. Four squares were defined as the center, and the 12 squares along the walls as the periphery. All open field tests were conducted under light intensity of 400 lx. The field was routinely cleaned with ethanol following each session. We scored the times of line crossings and the time spent in the center of the open field by the animals.

To further explore the effect of stimulation on locomotion, we performed an additional experiment with a different group of mice (*n* = 6 each group) in which mice explored the arena for a longer time (15 min). We scored the line crossings.

*Novelty-suppressed feeding (NSF) test.* After twenty-four hours food deprivation in the home cage, food pellets were placed in the center of an arena (50 × 50 cm) covered with clean bedding. The test was carried out during a 5 min period in a brightly illuminated (1400 lx) room. The mice (*n* = 10 each group) were positioned individually in a corner of the arena, and the latency to feed was recorded. Afterward, we measured the food consumed within 5 min after the allocation to their home cages.

*Elevated plus maze (EPM).* We used a device that included elevated and enclosed arms. The trials lasted 5 min. The following parameters were analyzed: open arm time, open arm entries made, closed arm time, latency to the first open arm entry, and number of open arm entries. A detailed description of the methodology and the apparatus was described elsewhere [25].

Tail *suspension test (TST).* The mice (*n* = 10 each group) were suspended with an adhesive piece of duct tape attached to a 30 cm long rigid tape which hung from a strong horizontal metal rod (*n* = 10 each group). The suspended mouse was video recorded for 6 min. An observer scored the total duration of a passive, “dead weight” hanging (immobility) [26]. Only the data scored during the last 4 min were analyzed.

*Forced swim test (FST).* The forced swim test was described before [27]. Mice (*n* = 10 each group) were tested for 6 min in a water-filled cylinder (25 °C). Only the data scored during the last 4 min were analyzed. The duration of immobility (the time during which mice made only the small movements necessary to keep their heads above water) was used in the analysis.

### 2.4. Hippocampal Progenitor Cell Proliferation

To label newly generated cells, the mice (*n* = 5 each group) were intraperitoneally injected with 200 mg/kg bromodeoxyuridine (BrdU, Sigma-Aldrich, USA) after the behavioral tests. Two hours after the injection, mice were anesthetized with sodium pentobarbital (30 mg/kg), sacrificed, transcardially perfused with ice-cold 1 mol/L PBS (pH 7.4), followed by ice-cold 4% paraformaldehyde in 1 mol/L PBS. Brain slices were immunostained (BrdU and doublecortin-DCX), examined, and quantified as previously described [25]. All samples were number-coded, and blinded analysis was performed.

### 2.5. Mature and Newly Generated Granule Cell Sholl Analysis

Golgi impregnation: under deep sedation, mice were sacrificed and transcardially perfused with 4% paraformaldehyde in PBS. Then, brains were postfixed (2% paraformaldehyde/2.5% glutaraldehyde in PBS) for >24 h and stained with FD Rapid GolgiStain Kit (FD NeuroTechnologies, Ellicott City, MD, USA). Briefly, brains were placed into solutions A and B for 2 weeks (room temperature) in the dark and for 48 h blocks in Solution C (4 °C). The samples were frozen in dry ice and were stored at −70 °C until sectioning. The samples were subjected to cryostat section to produce 100 μm sections, which were mounted in a slide. The slides were rinsed in distilled water and placed in solution containing silver nitrate (10 min). Sections were then cleared and covered with cover slips and examined [26].

Sholl analysis was performed to identify Golgi-stained and Doublecortin-positive (DCX^+^) granule cells with tertiary, relatively untruncated dendritic branches (*n* = 5 cells per brain, *n* = 5 each group) using camera lucida at 40× magnification (Neurolucida, Microbrightfield, Williston, Vermont). NeuroExplorer (Microbrightfield) was adopted to evaluate dendritic length and number of intersections (branch points) [28].

Golgi-stained and DCX^+^ granule cells with tertiary, relatively untruncated dendritic branches (*n* = 5 cells per brain, *n* = 5 each group) were traced using camera lucida at 40× magnification (Neurolucida, Microbrightfield, Williston, Vermont). Using NeuroExplorer (Microbrightfield), we calculated dendritic length and number of intersections (branch points). All samples were number-coded, and blinded analysis was performed [25].

### 2.6. Statistical Analysis

Group differences were analyzed with unpaired, two-tailed *t*-test. Dendritic complexity was assessed with a parametric two-way ANOVA (main effects: stimulation and radius). The data were analyzed with the Stat View statistical software package (SAS Institute Inc., Cary, NC, USA). *p* < 0.05 was considered statistically significant.

## 3. Results

### 3.1. 1-Hz rTMS Modulates Depression-Like Behaviors

We first tested mice in anxiety-like behavioral tests (OFT, NSF, EPM) to investigate possible effects caused by the 1 Hz-rTMS treatment. Normally, in the OF test anxious mice spend more time in the periphery than in the center of the testing arena. 1-Hz rTMS- and Sham-stimulated mice did not show an obvious difference in the amount of time spent in the center of the field (1-Hz rTMS: 17.3 ± 3.2 s; Sham: 18.8 ± 3.8 s, *p* = 0.6) and in the total number of crossings (1-Hz rTMS: 60.3 ± 6.9; Sham: 72.4 ± 7.2, *p* = 0.7). There was also no difference in the number of the crossings in separate experiments in which animals were given longer time to explore the arena (1-Hz rTMS: 202.6 ± 9.5; Sham: 194.7 ± 11.3, *p* = 0.47).

Anxiety was further evaluated by using the NSF and EPM tests. In the NSF test, anxiety level is indicated by a shorter latency to consume a familiar food in a novel environment. Latency to feeding (1-Hz rTMS: 78.1 ± 10.5 s; Sham: 83.4 ± 10.2 s, *p* = 0.39) and home cage food consumption (1-Hz rTMS: 3.8 ± 1.1 g; Sham: 3.89 ± 0.9 g, *p* = 0.6) were not different between groups.

In the EPM, mice that exhibited higher anxiety levels spent less time in the open arms of the maze. The latency to opened arm entry (1-Hz rTMS: 9.7 ± 1.9 s; Sham: 10.1 ± 2.1 s, *p* = 0.8), the percentage of time in the open arm (1-Hz rTMS: 55.3 ± 5.5%; Sham: 52.4 ± 5.9%, *p* = 0.618), and the percentage of open arm entry (1-Hz rTMS: 60.7 ± 6.3%; Sham: 57.4 ± 6 %, *p* = 0.7) were not different between groups.

**Figure 2:**
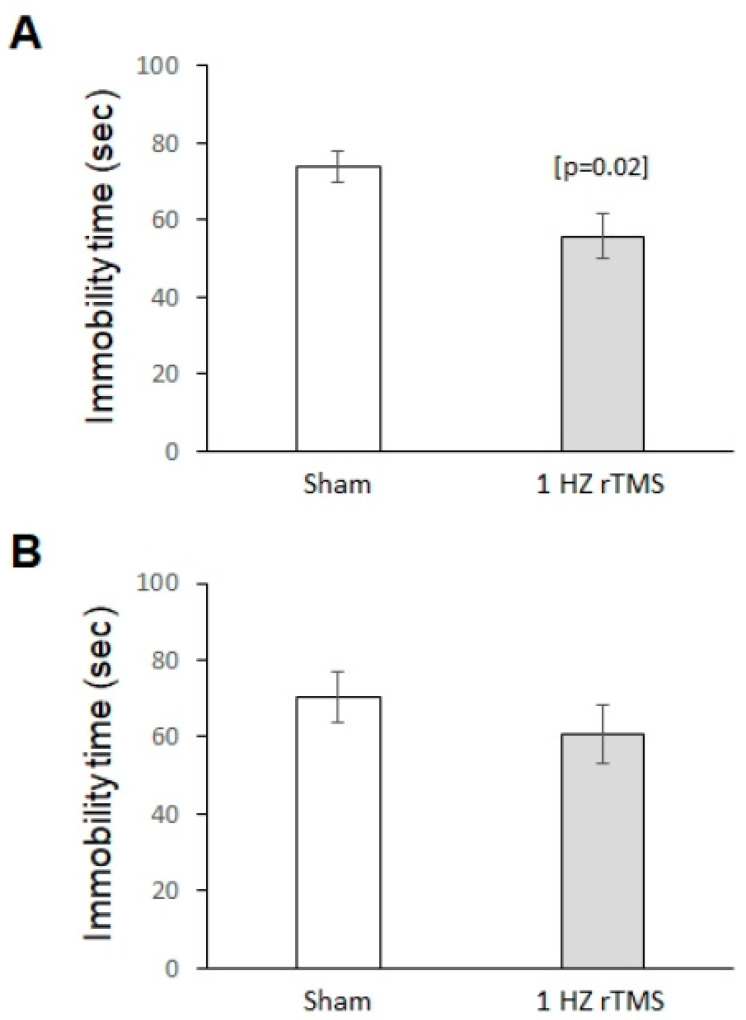
1-Hz rTMS modulates depression-like behavior. (**A**) Compared with Sham-stimulated mice, 1-Hz rTMS-treated mice showed reduced depression-like behavior in the tail suspension test. (**B**) In the forced swim test, immobility time was similar between the two groups. Data are reported as mean ± SE (*n* = 10 in both groups). *p* < 0.05.

We then investigated the effect of treatment on depression-like behaviors using TST and FST. In the TST, 1-Hz rTMS significantly decreased immobility time (*p* = 0.02) (Figure 2A). On the contrary, there were no differences in the immobility time in the FST between groups (*p* = 0.37) (Figure 2B).

### 3.2. 1-Hz rTMS Increases Mature Granule Cell Dendritic Complexity in Mice

**Figure 3:**
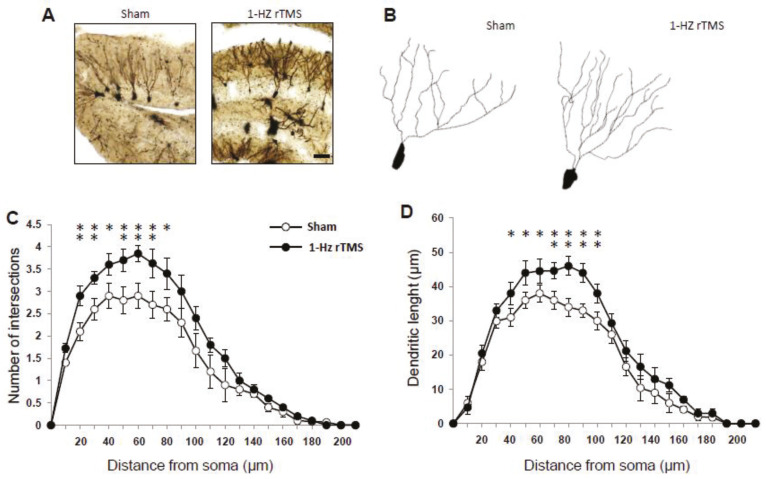
1-Hz rTMS modulates mature granule cells dendritic morphology. Mature granule cells showed significant higher number of intersections (**A**) and dendritic length (**B**) in the 1-Hz rTMS mice compared with the Sham-stimulated mice. Sholl’s analysis indicated that the 1-Hz rTMS group showed increased number of intersections (**C**) and increased dendritic length (**D**). Data are reported as mean ± SE, *n* = 5 in both groups; 5 cells each mouse. * *p* < 0.05, ** *p* < 0.01.

We then investigated mature granule cell dendritic complexity. Figure 3 illustrates the Sholl analysis of dendritic length (A) and dendritic intersections (B). Both parameters were affected by the treatment (main effect stimulation: F_(1168)_ = 29.4, *p* < 0.0001; radius: F_(20,168)_ = 31.1, *p* < 0.0001; stimulation x radius interaction: F_(1138)_ = 9.6, *p* < 0.0001) and indicate that the stimulation protocol increased mature granule cell complexity.

### 3.3. 1-Hz rTMS Modulates Newly Generated Granule Cell Dendritic Complexity in Mice

**Figure 4:**
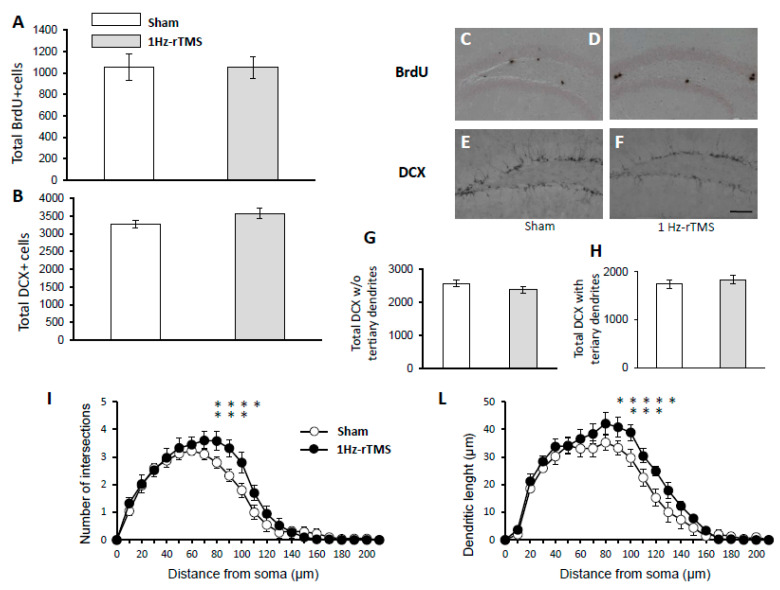
1-Hz rTMS, neurogenesis, and dendritic complexity of newly generated neurons. (**A**,**B**) The number of thymidine analogue bromodeoxuridine (BrdU)-incorporated cells was not significantly different in the granular cell layer (GL) between Sham- and 1Hz- rTMS-stimulated mice. (**C**–**F**) Representative images of BrdU and Doublecortin (DcX)+ neurons. Scale bar, 200 μm. Quantification of DcX+ cells without (**G**) and with tertiary dendrites (**H**). (**I**,**L**) Dendritic length and intersections in newly generated granule cell are increased in the 1-Hz rTMS-treated mice. Data are reported as mean ± SE, *n* = 5 in both groups; 5 cells each mouse. * *p* < 0.05. ** *p* < 0.01.

To evaluate progenitor cell proliferation in the DG, BrdU was injected intraperitoneally in both Sham- and 1-Hz rTMS-stimulated mice and the BrdU-labeled cells were quantified in DG 2 h after the injection. The rTMS-stimulated mice displayed a comparable number of hippocampal BrdU-positive cells (*p* = 0.2) (Figure 4E). There was no between-group difference in the number of Dcx-positive cell (*p* = 0.4) (Figure 4B), DCX^+^ cells without tertiary dendritic processes (t = 0.9, *p* = 0.38) (Figure 4G), and with tertiary dendritic processes (*p* = 0.09) (Figure 4H). Furthermore, compared with Sham-stimulated mice, 1-Hz rTMS-stimulated mice showed changes in dendritic length (main effect stimulation: F_(1168)_ = 14.3, *p* = 0.1; radius: F_(20,168)_ = 14.3, *p* = 0.09; stimulation x radius interaction: F_(1138)_ = 18.3, *p* = 0.02) (Figure 4I) and number of intersections (stimulation: F_(1120)_ = 8.5 *p* = 0.13; radius: F_(14,120)_ = 0.81, *p* = 0.2; stimulation x radius interaction: F_(1120)_ = 0.92, *p* = 0.3) in DCX^+^ cells with tertiary dendrites (Figure 4L).

Overall, the observed changes demonstrate that 1-Hz rTMS modulates both mature and newly generated granule cell dendritic complexity.

## 4. Discussion

In our model, 1-Hz rTMS modulates depression-like behavior in the TST in male mice. Furthermore, our results indicate a modulatory effect of 1-Hz rTMS on dendritic morphology in both mature and newly generated granule cells. However, the treatment did not affect the proliferation of adult DG progenitor cells.

One-Hz rTMS-treated mice exhibited less immobility when challenged in an inescapable situation such as the TST, while the anxiety-like behavior tests (OF, NSF, and EPM) were not modulated by the stimulation protocol. These results indicate that 1-Hz rTMS treatment does not increase sensitivity to novelty and decreases behavioral despair in mice. Moreover, our results are consistent with a previous study that demonstrated similar antidepressant-like effect of the LF rTMS on rats in the FST [29]. Given that the number of crossings in the OF was not different between groups (as demonstrated with two OF paradigms), it is conceivable that the TST results are not confounded by baseline differences in locomotor activity. Future studies using computerized systems are needed to evaluate the effect of the treatment on total mice ambulatory distances. It should be noted that previous studies comparing TST and FST suggested that FST is a higher stressor involving different neuronal systems [30], neurotransmitters [31], and neurotrophic factors [32,33]. Thus, one compelling interpretation of our results is that the stressful experimental protocol (restrain, TMS noise) might have played a role in determining a different modulation of the FST and TST. Furthermore, the greater sensitivity of the TST in detecting the antidepressant-like effects of 1-Hz rTMS might be strain-specific (i.e., 129/SvEv strain) [34] and/or gender-specific (male mice). Future studies using different strains and female mice are needed to fully address the behavioral effect of 1-Hz rTMS. Furthermore, although the 129/SvEv mouse strain is prone to anxiety [23] and shows longer immobility during behavioral despair tests [24], our data should be confirmed and expanded by using different animal models of anxiety and depression and using a repeated-measure study design.

The main goal of this study was to examine the effect 1-Hz rTMS on granule cell dendritic morphology. It was demonstrated that long-term potentiation (LTP) is associated with increased dendritic plasticity [35,36] while long-term depression (LTD) induces opposite changes [37,38]. In humans, 1-Hz rTMS treatment produces a decrease in motor-evoked potential amplitude (MEP) [39]; these effects are thought to depend upon LTD-like plasticity at cortical synapses [40]. Thus, the increase in dendritic complexity we detected might be consistent with a plastic effect [41]. It is also conceivable that the changes in dendritic plasticity might be a consequence of the repeated activation of these synapses. Dendrites are highly dynamic structures and change their structure and complexity in response to the stimuli received [42]. According to this hypothesis, our results could represent a form of activity-dependent plasticity [43,44,45] and expand a previous finding indicating that LF rTMS treatment increased the hippocampal synapses density [46]. It is likely that these changes may affect the information processing at the circuit level, influencing, in this way, complex behaviors [47]. Furthermore, although granule cells are GABAergic, it needs to be demonstrated in future electrophysiological experiments whether these structural changes modulate inhibitory or excitatory circuits.

We showed that 1-Hz rTMS treatment enhances dendritic plasticity without modifying DG progenitor cell proliferation. Previous studies provided evidence linking adult hippocampal neurogenesis with antidepressants action in specific rodent behavioral tests [48]. Furthermore, DCX^+^ cells displayed more complex dendritic arborization after chronic fluoxetine treatment [19] and seizure induction, (a condition that mediates therapeutic effects during ECT [49]). Taken together, these data suggest that the dendritic plasticity of newly generated granule cells might play a role in mediating antidepressant effects of 1-Hz rTMS.

The study has some limitations. It is likely that, due to the nonfocal nature of rTMS using the rodent coil in awake mice, our stimulation protocol might have induced similar plastic changes in different brain areas (bilateral stimulation). Alternative experimental protocols might provide a more focal stimulation [50], but the repeated anesthesia might interfere with the development of the structural plastic changes and, eventually, with the behavioral gain. Furthermore, the stimulation procedure could be optimized using mathematical modeling and adjusting the stimulator intensity during the sham condition to ensure exposure to a “click” of the same intensity during the experimental conditions. Thus, due to these limitations, the specific contribution of 1-HZ rTMS to the hippocampal dendritic plasticity and emotional behaviors need to be further investigated in future experiments.

Future studies using different parameters (intensity, duration, handling), using mice with different genetic background, and including both male and female mice are needed to confirm the generalizability of our results.

## 5. Conclusions

In summary, our data showed that 1-Hz rTMS induced antidepressant-like effects on mice without altering anxiety-like behaviors. Furthermore, the treatment increases both newly generated and mature granule cell dendritic complexity. We cannot speculate for a causal link between changes in hippocampal morphology and antidepressant effects. Nevertheless, these results provide critical evidence indicating that 1-Hz rTMS promotes dendritic plastic changes in circuits that regulate chronic stress and depression.
